# Micro-optical lens array for fluorescence detection in droplet-based microfluidics†
†Electronic supplementary information (ESI) available: Supplementary Figures (S1 and S2). Supplementary movie 01: movie recorded by a high-speed camera without backlight illumination. Supplementary movie 02: movie recorded under similar conditions as movie 01 with backlight illumination. Supplementary movie 03: captured using a standard camera through the eyepiece of the microscope at a frame rate of 23 fps. See DOI: 10.1039/c3lc41329bClick here for additional data file.



**DOI:** 10.1039/c3lc41329b

**Published:** 2013-03-01

**Authors:** Jiseok Lim, Philipp Gruner, Manfred Konrad, Jean-Christophe Baret

**Affiliations:** a Max Planck Institute for Dynamics and Self-Organization , Am Fassberg 17 , 37077 Goettingen , Germany . Email: jean-christophe.baret@ds.mpg.de; b Max Planck Institute for Biophysical Chemistry , Am Fassberg 11 , 37077 Goettingen , Germany

## Abstract

We demonstrate the design and integration of droplet-based microfluidic devices with microoptical element arrays for enhanced detection of fluorescent signals. We show that the integration of microlenses and mirror surfaces in these devices results in an 8-fold increase in the fluorescence signal and in improved spatial resolution. Using an array of microlenses, massively parallel detection of droplets containing fluorescent dyes was achieved, leading to detection throughputs of about 2000 droplets per second and per lens, parallelized over 625 measurement points.

## Abstract 2

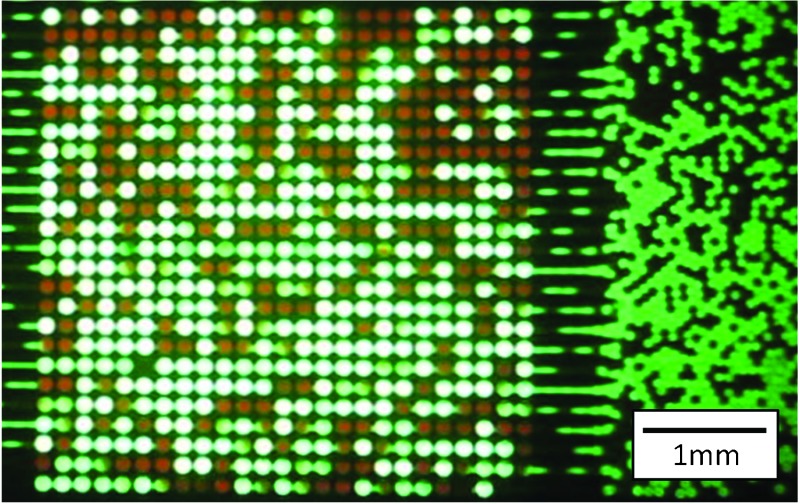
We demonstrate the design and integration of droplet-based microfluidic devices with microoptical element arrays for enhanced detection of fluorescent signals.

Droplet-based microfluidic systems are promising platforms for high-throughput screening applications.^1,2^ The versatile encapsulation of various types of biomaterials, such as proteins, nucleic acids, single genes or cells makes droplets perfectly suitable as a tool for miniaturized and automated enzymatic assays,^3^ cell screening,^4–8^ molecular cancer diagnostics^9–11^ or drug screening platforms.^12,13^ The success of the technology relies on the very high-throughput of each individual step of droplet manipulation, which reaches several kilohertz for droplet production, re-injection, fission or fusion.^14^ Droplet production rates larger than 10 kHz have been reached and can in principle be further increased by parallelization. With increased throughput in droplet production, analytical tools have to be developed accordingly, to avoid the analysis of single droplets becoming the bottleneck of the operation. In order to extract analytical information, fluorescence intensity measurements are commonly performed as a convenient, non-destructive, rapid and sensitive method. Signal detection is usually done by photomultipliers, photodiodes or processing of images generated by standard or high-speed cameras.^7,13,16,28^ These conventional detection systems have limitations when applied for massively parallel detection: (i) optical systems with a high numerical aperture such as photomultipliers can offer sufficient sensitivity, but a multi-point measurement is difficult to achieve; (ii) small numerical aperture objectives with a large field of view allow for parallelization, but their optical sensitivity is limited.

Up to now, parallelization of optical detection has been the bottleneck for high-throughput screening because of the trade-off between field of view and sensitivity. Only a very limited range of solutions is available.^16,27^ In this paper, we describe an integrated micro-optical system for droplet-based microfluidics.

We make use of wide field illumination to obtain measurements on wide areas, providing parallelization, while a micro-optical lens array is integrated to enhance imaging resolution and sensitivity. In addition, a mirror structure on the channel wall induces optical resonance to enhance the signals. The fluorescent signal intensity is improved without sacrificing of the field of view due to the overall numerical aperture of the optical system. The schematic diagram of the device is shown in Fig. 1.

**Fig. 1:**
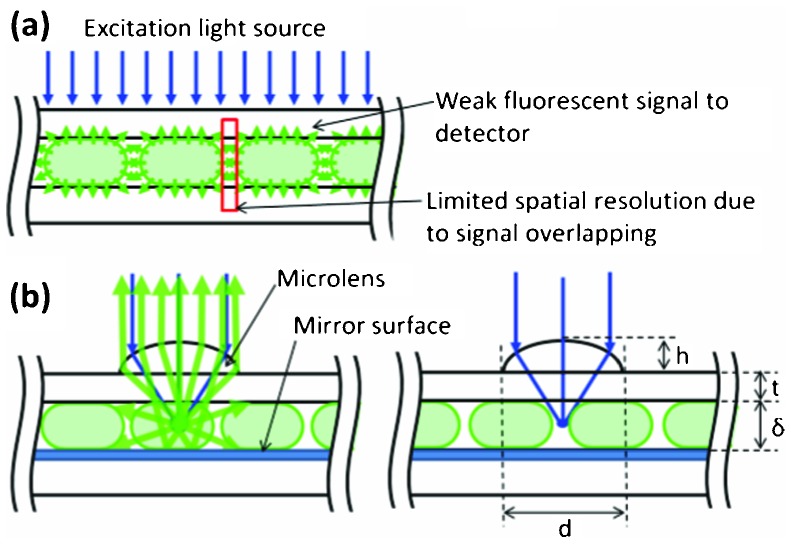
Limitations of conventional droplet based microfluidic device (a). Schematic diagram of the microoptics integrated droplet-based microfluidic device (b). The microlens improves the excitation light intensity and fluorescent signal collection. The metallic mirror surface on the channel structure enables the fluorescent signal collection. Due to the localized beam intensity at the spot, spatial resolution is improved.

The microlens with 25 × 25 array dimension was designed for generating an optical spot in the droplet through a 2 inch cover glass with thickness of *t* = 150∼160 μm (No.1 cover slip). The back focal length of the microlens (*f*_b_) should be *t* < *f*_b_ < *t* + *δ*, where *δ* is the channel height, to generate a focal point in the channel. The diameter (*d*) and sag (*h*) of the lens were selected as 120 μm and 20 μm respectively. The radius of curvature (*R*) is determined from spherical cap approximation *R* = (*d* ^2^ + 4*h* ^2^)/8*h* = 100 μm.^23^ The back focal length (*f*_b_) and numerical aperture (NA) were then calculated by the following equations:^15^
1
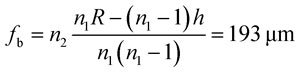

2
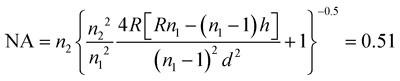
where *n*_1_ = 1.67 and *n*_2_ = 1.47 are the refractive indexes for microlens and glass substrate, respectively. To ensure an identical pitch of lenses and channels, the lateral shrinkage of PolyDiMethylSiloxane (PDMS) during polymerization had to be considered. A value of 1.4%, measured by using a microscaler, was taken into account for the design of the lens array. The microlens array was fabricated by a thermal reflow process^23–26^ using positive photoresist AZ9260 which is composed of the thermoplastic polymer propylene glycol monomethyl ether acetate. After fabricating the cylindrical pedestal structure on a glass substrate, it was thermally treated up to glass transition temperature (150 °C). In this manner the photoresist shape could be converted into a spherical cap by surface tension. To construct the designed microlens with sag of 20.0 μm, we first fabricated the pedestal structure with thickness of 11.8 μm. After 1 min thermal treatment at 150 °C, the designed shape was generated.

The channel structure of the microfluidic device was replicated in PDMS from an SU-8 (negative) photoresist with thickness of 35 μm; it was manufactured using a standard UV-photolithography process.^17^ Mirror structures were deposited on the channel walls by metal-evaporation processes. Gold and silver double layers were deposited by thermal evaporation, yielding thicknesses of 100 nm and 200 nm, respectively. The mirror structure is designed to have (i) a reasonable reflectance at the wavelengths of excitation and emission, (ii) reversible adhesion property with PDMS to withstand fluid flows in the channel, and at the same time to be peeled off during the manufacturing steps. Gold was selected as the first layer because of its favorable adhesion properties with PDMS. As its reflectance at 490–520 nm (practical wavelength range for fluorescence excitation and emission) is only 35∼60%, a silver layer as reflecting surface was deposited on top, because of its high reflectivity (>90%) in the desired wavelength range. Prior to glass bonding, the metallic layer on the PDMS surface was removed using adhesive tape (3M).

Finally the glass substrate carrying the microlens array was aligned using a stereomicroscope equipped with a 4-axis (*XYZ-θ*) stage with the prepared PDMS structure and bonded by using standard oxygen plasma treatment (Fig. 2).

**Fig. 2:**
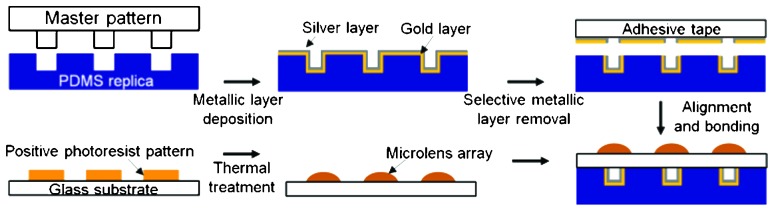
Fabrication process flow of the microoptics integrated microfluidics: top: microfluidic channels; bottom: micro-optical lens array.

Two different types of droplet-based microfluidic chips were designed and fabricated: a device composed of a 10 cm-delay line structure and a 25 parallel channel structure for re-injection (Supplementary Fig. 1†). Droplets of phosphate-buffered saline (PBS) containing varying concentrations of fluorescein or resorufin were produced by flow focusing^18^ in a fluorocarbon oil (HFE-7500) with 0.5% w/w PEG-PFPE block-copolymer surfactant produced according to standard protocols,^19^ used to prevent droplet coalescence.^20^ Flow rates were controlled by syringe pumps (neMESYS, Cetoni). Droplets were produced at a rate of ∼0.8 kHz using an aqueous phase flow rate of 5 μL min^–1^, and an oil-phase flow rate of 20 μL min^–1^, unless stated otherwise in text.

A high power LED light source (CoolLED pE-2, blue LED) was used for excitation. The high-speed camera (Phantom v210) was assembled on an inverted microscope (Olympus IX71) with 2-fold magnification objective lens (NA of 0.06). A dichroic mirror (AHF) mounted on a filter cube was used to split excitation and emission. The high-speed camera was operated for data acquisition. A standard video camera was used to record movies from the microscope eye-piece.

We first used Device 1 to characterize the optical efficiency of our system. Fluorescence signals were recorded using the high-speed camera. The inset of Fig. 3(a) shows a typical frame of the movie as recorded on the high-speed camera at a frame rate of 4000 fps and exposure time of 250 μs, using a fluorescein concentration of 100 μM.

**Fig. 3:**
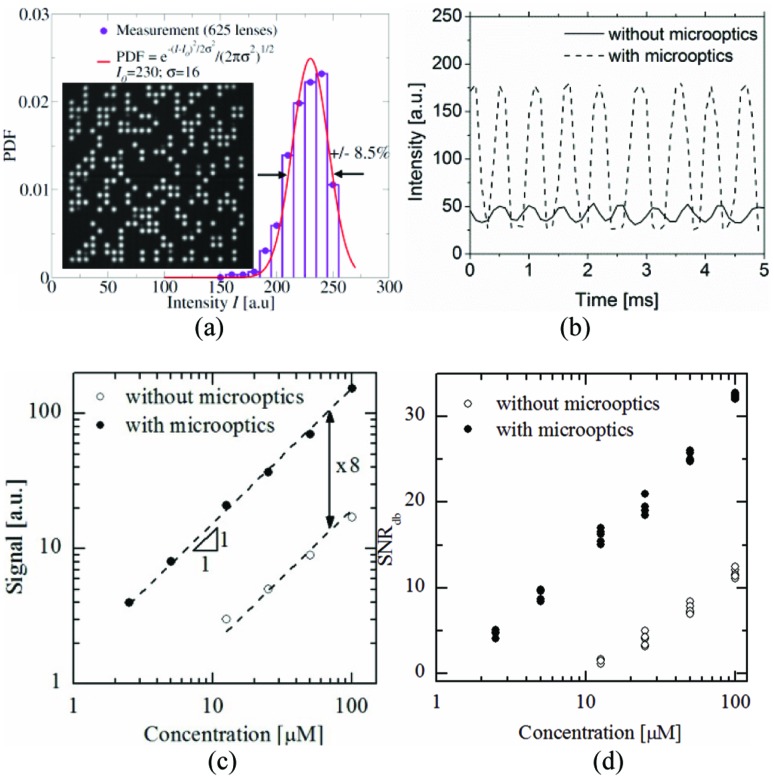
(a) Signal measured over 625 lenses (100 μM fluorescein, see Supplementary Movies 01 and 02†). Inset: Example of one typical frame. (b) Fluorescent signal comparison between conventional and microoptics integrated devices. (c) The fluorescent signal was enhanced ∼8 times; the signal from 10 μM fluorescein detected by the developed device is similar to that obtained from 100 μM using a conventional chip. (d) SNR comparison between newly developed and conventional devices in decibel scale.

A white spot indicates a droplet flowing below the corresponding lens, while in the absence of a droplet, the signal is dark (Fig. 3(a) and see Supplementary Movies 01 and 02†). The array structure of the micro-lens is clearly visible. The captured movie was post-processed as an averaged intensity of 3 × 3 pixels centered in the middle of each lens and was recorded as a single data point. The maximum throughput with this system was reached at flow rates of 10 μL min^–1^ (aqueous phase) and 40 μL min^–1^ (oil phase). The measurement throughput then was ∼2000 drops per second and per lens (Fig. 3(b)) with up to 625 measuring points. The uniformity of our measured signals over the 625 microlenses showed a deviation of ±8.5% (Fig. 3(a)). The high-speed camera was then operated with a frame rate of 10 000 fps and 100 μs exposure time. Under these conditions, each droplet is measured at about 5 detection points which clearly sets the throughput limit.

In order to quantify the observed signal enhancement, we systematically measured the fluorescent signals at various fluorescein concentrations from 2.5 μM to 100 μM, and compared the results with a device devoid of lenses and mirror. We found that fluorescence signals were increased approximately 8 times (Fig. 3(d)). Of special interest is the gain of sensitivity below 25 μM where the signal without lens is hidden in the noise. Our device provides means to detect fluorescein concentrations down to 2.5 μM. The detection limit can be decreased using for example a higher power light source excitation. Alternatively, reducing droplet throughput and increasing the lens density can improve the sensitivity without loss of information. The signal-to-noise ratio in dB was calculated according to standard methods^21^
3
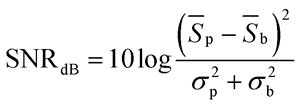
where *S*_p_ and *S*_b_ are the average peak-fluorescence and background signals, respectively, and *σ*_p_ and *σ*_b_ are the deviations of each signal. Fig. 3(c) shows the quantitative gain in sensitivity: about 8-fold increase in signal and 20 db in signal-to-noise ratio. The mirror fabricated by metallic layer deposition improves the efficiency of excitation and collecting fluorescence signal. In the case of transparent PDMS, the light for excitation will simply penetrate through the droplet, and the emission rays from the droplet will equally radiate to any direction. The mirror structure will provide a longer optical pathway of the exciting light through the droplet due to the resonating effect, and the extraction efficiency of the fluorescent light will be guided to the detector more efficiently. We actually compared the signals from a chip with and without the mirror and measured 35∼40% signal increase with the mirror.

Device 2 was used to investigate the spatial resolution enhancement. Droplets containing 100 μM and 50 μM of fluorescein were first prepared with a dual droplet maker^22^ and re-injected at a flow rate of 100 μL min^–1^. The re-injection procedure provided very small droplet spacing. The smallest gap between droplets was less than 10 μm. Fig. 4 shows the comparison between the micro-optical lens array and the conventional system.

**Fig. 4:**
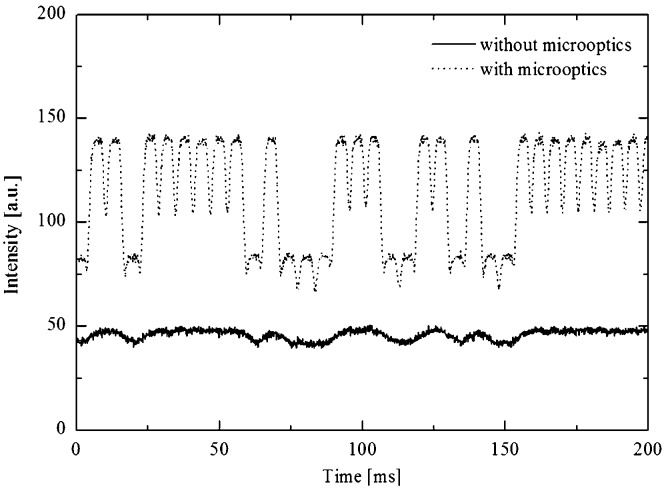
Measured fluorescent signals resulting from the microoptics integrated system as compared to a conventional device, using droplets containing 100 μM (big peaks) and 50 μM (small peaks) fluorescein.

Here the improved resolution coupled to the increase of the signal enables to discriminate the succession of droplets. Such tool would be of interest for the measurement of droplets on re-injected emulsions, for example in applications for medical diagnostics.^9^ For clarity, we show only one measurement point, but signal detection is indeed parallelized over 625 measurement points for achieving increased throughput.

The experiments presented above involve the use of a monochrome high-speed camera, which restricts the intensity measurements to a single color. Though this camera already is an expensive piece of equipment, a color version would further increase the cost of the detection system. Such equipment may be admissible for certain types of diagnostics platforms, but low cost alternatives would be beneficial. As a proof of principle, we demonstrated here the use of a standard custom video camera (23 fps, color). This conventional camera served for capturing movies through the microscope eyepiece. An emulsion containing droplets of fluorescein (100 μM; green fluorescence) or resorufin (100 μM; red fluorescence) was re-injected into the device at a flow rate of 50 μL min^–1^. In areas not covered by the micro-optic lenses, the red fluorescence of resorufin was barely visible, but in the region where droplets flow under the lens, the detector clearly displays a red signal (Supplementary Fig. 2 and Supplementary Movie 03†).

Obviously, the throughput was decreased here to match the camera frame rate but the parallelized analysis still maintains a decent throughput rate of 500 drops per second (20 drops per second and per channel) with 625 measuring points. The throughput can be easily multiplied by a factor of 4 which is achievable by optimizing the channel design and the re-injection conditions. Alignment strategies described in previous publications^16^ could also be used to guarantee one measurement per channel, but the device presented here demonstrates the capabilities of the micro-optic array to gain sensitivity, intensity, and spatial resolution in applications where low cost is crucial. Due to the fixed microlens array integrated region and the order of the signal, simple image processing filters can be used to quantitatively analyze the emulsion.

We demonstrate here the integration of a micro-optical lens array with droplet-based microfluidics. The microoptics can be combined with any type of droplet-based microfluidics using simple alignment procedures. Such a combination provides increased sensitivity and resolution through the focusing of light by a single element while increased throughput is achieved by integration of the lenses in an array. We believe that our system will allow for application not only in high-throughput droplet analysis but also for cells and fluorescent particles as it can provide improved fluorescence signals, and a potentially significant decrease of analytical costs.
