# Supplementary material for: Micro-optical lens array for fluorescence detection in droplet-based microfluidics
Source: Lab Chip. 2013 Mar 1;13(8):1472–5. doi: 10.1039/c3lc41329b (PMC3697795; doi:10.1039/c3lc41329b)
Supplement: Supplementary file 1 [file LC-013-c3lc41329b-s001.pdf]

This journal is © The Royal Society of Chemistry 2013

Lab on a Chip
Supplementary Information

Micro-optical lens array for fluorescence detection in droplet-based microfluidics

Jiseok Lim, Philipp Gruner, Manfred Konrad and Jean-Christophe Baret

Supplementary Figure 1: Photograph and microscopic image at the microoptical region of interest for the Two different types of droplet-based microfluidic chips we designed and fabricated: a device composed of a 10 cm delay line structure (a) and a 25 parallel channel structure for re-injection (b).

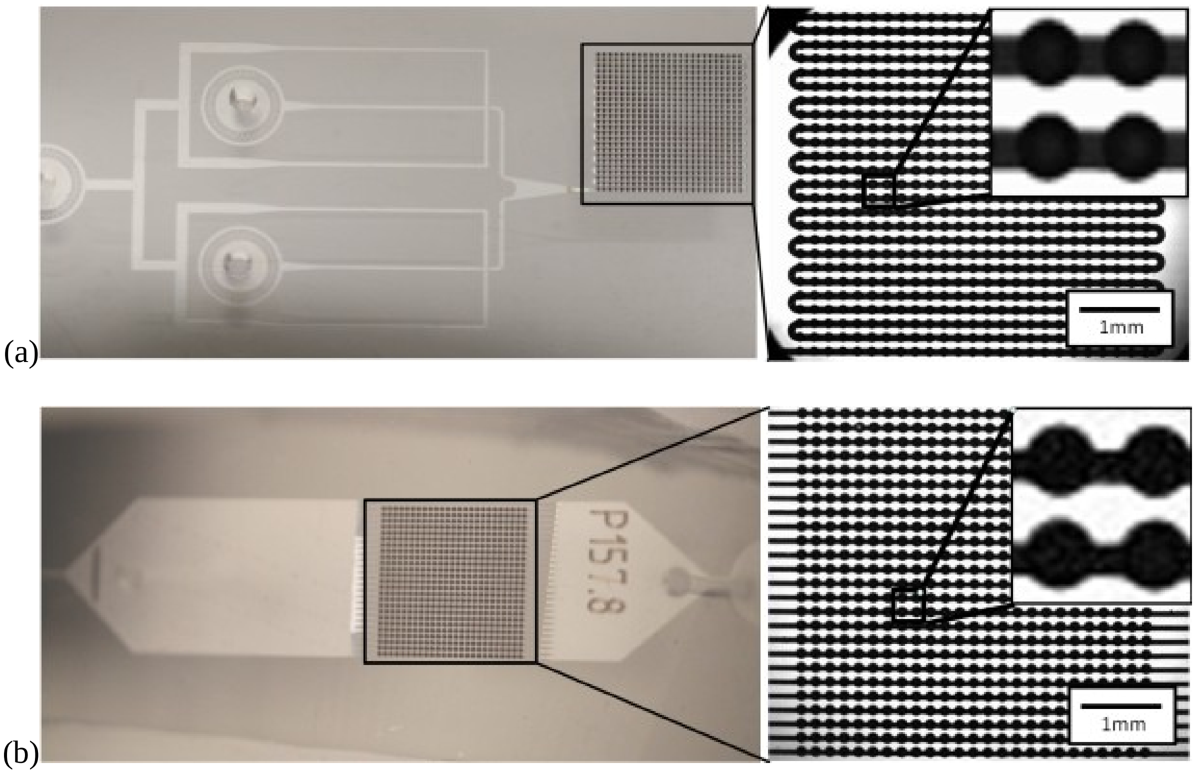

(a)

(b)

This journal is © The Royal Society of Chemistry 2013

Supplementary Figure 2: Imaging through the microscope eyepiece with two different fluorescent dyes (100 mM of resorufin and 100 mM of fluorescein – see supplementary movie 033). Droplets flow from right to left. The droplets containing resorufin can only be detected in the presence of microlenses. See also **Supplementary movie 03**.

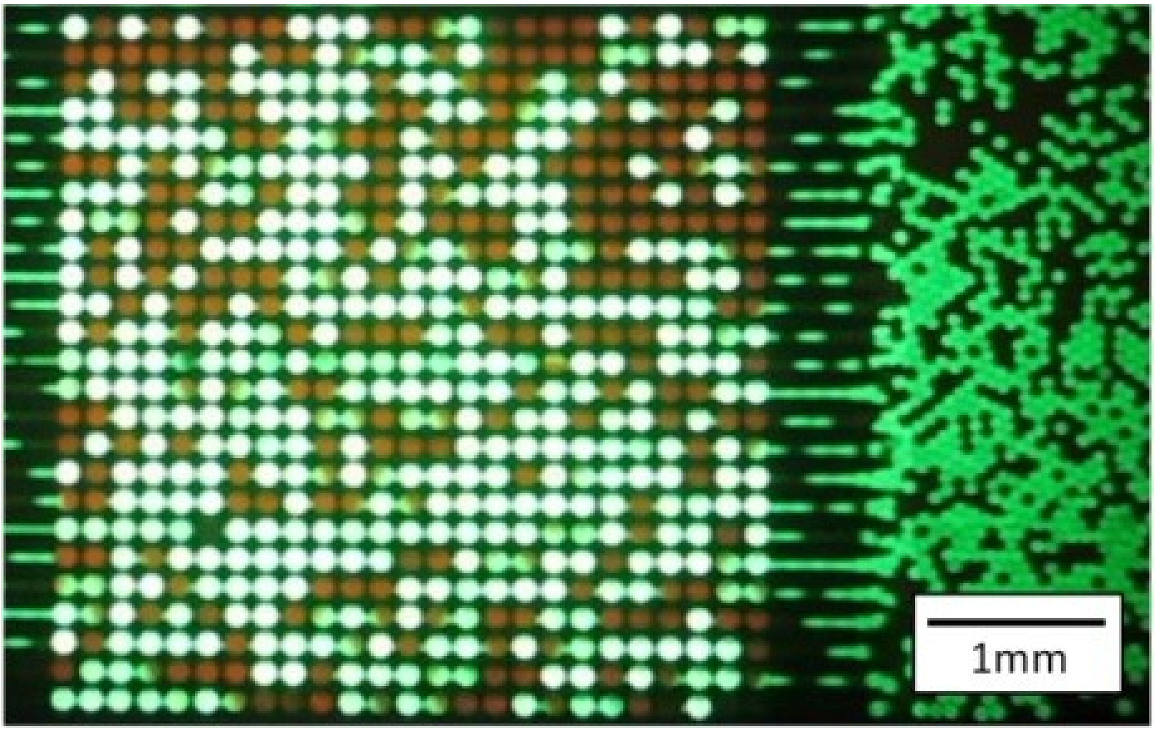

Supplementary Movies:

Supplementary movie 01:
movie of droplet flowing under the lense array recorded by a high speed camera without backlight illumination.

Supplementary movie 02:
movie recorded under similar conditions as movie 01 with backlight illumination.

Supplementary movie 03:
movie captured using a standard camera through the eyepiece of the microscope at a frame rate of 23 fps.
